# Incidence rate of hemorrhagic fever with renal syndrome complicated with acute pancreatitis: a meta-analysis

**DOI:** 10.3389/fmed.2024.1442276

**Published:** 2024-10-22

**Authors:** Zhenzhen Ye, Xiaoqing Liu, Sheng Ding, Ling Lu, Tianchen Zhang, Wenfang Zhou, Yonghai Dong

**Affiliations:** ^1^Youth Research and Innovation Team of Jiangxi Provincial Center for Disease Control and Prevention, Nanchang, Jiangxi, China; ^2^Prevention Rural Environmental Health Technical Guidance Center of Jiangxi Provincial Center for Disease Control, Nanchang, Jiangxi, China; ^3^Jiangxi Province Key Laboratory of Major Epidemic Prevention and Control, Nanchang, Jiangxi, China

**Keywords:** acute pancreatitis, hemorrhagic fever with renal syndrome, meta-analysis, mortality, HFRS

## Abstract

**Background:**

Acute pancreatitis (AP) is a rare but serious complication in patients diagnosed with hemorrhagic fever with renal syndrome (HFRS). When AP complicates HFRS, the clinical outcome significantly worsens and the risk of mortality increases. However, the incidence of AP in HFRS patients and its associated mortality risk remain unclear. To address this knowledge gap, we conducted a meta-analysis to determine the AP incidence rate in HFRS patients and assess the impact of AP on mortality in these patients.

**Methods:**

We systematically searched seven databases (PubMed, Web of Science, EMBase, Sinomed, Chinese National Knowledge Infrastructure, WanFang Data, and Chongqing VIP) for relevant studies on HFRS complicated by AP. The studies were selected using predefined inclusion and exclusion criteria based on the Population, Intervention, Comparison, Outcome, and Study design principle. Two independent reviewers screened the studies, and the quality of the included studies was assessed using the Agency for Healthcare Research and Quality and the Newcastle-Ottawa Evaluation Scale (NOS).

**Results:**

In total, 11 studies, encompassing 1,218 HFRS patients, met the inclusion criteria. The overall incidence of HFRS complicated by AP was 8.5% (95% *CI* for *r* 5.9–11.1%). The HFRS patients with AP had a significantly higher risk of mortality than those without AP (*OR* = 3.668, 95% *CI* for *OR* 1.112–12.031). No statistically significant differences were observed in the subgroup and meta-regression analyses.

**Conclusion:**

Although the incidence of AP in HFRS patients is not high, it significantly increases the risk of mortality in these patients. Future large-scale prospective studies are required to further validate these findings.

## 1 Introduction

Hemorrhagic fever with renal syndrome (HFRS) is a rodent-borne zoonotic disease caused by hantaviruses (1). Initially classified under the genus Hantavirus within the Bunyaviridae family, the latest taxonomy now places hantaviruses responsible for HFRS under the genus Orthohantavirus of the Hantaviridae family within the order Bunyaviridae (2). Humans are typically infected through the inhalation of aerosols formed from rodent excretions or secretions contaminated with hantavirus or by ingesting contaminated food (3, 4). Suspected cases of HFRS were first reported in Russia, Japan, and China in the 1920s. However, it was not until the Korean War in the 1950s, when more than 3,000 United Nations soldiers fell ill, that the disease garnered widespread attention (5–7). Currently, more than 100,000 cases of HFRS are estimated to be reported globally each year (8). Because hantavirus infections go unreported in many countries, the actual number of HFRS cases may be higher (9). HFRS has a global distribution, with Europe and Asia being the primary areas of prevalence (10, 11). According to reports, from 2000 to 2017, Russia reported an HFRS incidence of 4.9 per 100,000 people; in China, the rate was 0.9 per 100,000 between 2004 and 2021, while South Korea reported an incidence of 0.8 per 100,000 from 2001 to 2010 (12–14).

The case fatality rate of HFRS varies widely, ranging from 1 to 15% (15). The mortality risk increases substantially when HFRS is complicated by other conditions (16, 17). Common complications in HFRS patients include cerebral edema, pulmonary edema, luminal hemorrhage, and infections. Although less common, acute pancreatitis (AP) has also been reported in HFRS patients. AP is a severe condition characterized by inflammation of the pancreas, often leading to multiple organ failure and death if not diagnosed and treated promptly (18). When HFRS patients develop AP, their condition becomes far more complex and dangerous, with the potential for rapid deterioration and an elevated risk of mortality (19). In Guo Q’s study (20), the fatality rate of patients with HFRS complicated by AP was 24.1%, whereas that of HFRS patients without AP was only 3.5%. This underscores the significant risk that AP poses to HFRS patients, highlighting the significance of timely diagnosis and treatment of AP in HFRS patients.

Currently, systematic evaluation of the incidence of AP in HFRS patients has been insufficient, which negatively affects the clinical treatment of these patients. Fan H (21) reported an incidence of 7.7% for HFRS combined with AP, while Xia Y (22) reported a considerably higher incidence of 18.3%. These discrepancies are likely due to differences in sample size, study design, and geographic location. We hypothesize that HFRS patients with AP will have a higher mortality rate than those without AP and that the incidence of AP in HFRS patients may vary depending on regional factors, sample size, and study methodology. To understand the incidence of AP in HFRS patients and its impact on mortality, we conducted a meta-analysis based on published studies examining the association of HFRS with AP. The objectives of our meta-analysis are to provide a comprehensive analysis of the present study and to offer insights that can inform clinical diagnosis and treatment strategies for HFRS patients with this complication.

Although some studies have explored HFRS and its complications, including AP, a clear understanding of the incidence and associated mortality risk remains elusive. The variability in study methodologies and the absence of a synthesized analysis across different populations and geographic regions have contributed to this gap in knowledge. Our study addresses this limitation by conducting a systematic meta-analysis, offering a novel approach to evaluate these factors comprehensively—a method that has been unexplored in current literature.

## 2 Materials and methods

### 2.1 Search strategy

A comprehensive literature search was conducted across multiple databases, namely PubMed, Web of Science, EMBase, Sinomed, Chinese National Knowledge Infrastructure, WanFang Data, and Chongqing VIP, between March and September 2024 to identify all relevant studies on HFRS complicated by AP. The search terms used were a combination of keywords related to HFRS and AP: (“Hemorrhagic Fever with Renal Syndrome” OR “HFRS” OR “Epidemic Hemorrhagic Fever” OR “Nephropathia Epidemica” OR “Hemorrhagic Nephroso Nephritis” OR “Korean Hemorrhagic Fever”) AND (“Pancreatitis” OR “Acute Pancreatitis” OR “Complications” OR “Coexistent disease” OR “Prognosis” OR “Prognostic Factor”). Two independent reviewers screened the literature, beginning with titles and abstracts, followed by a full-text review. Additionally, references of the included studies were examined for further relevant studies. No language restrictions were applied during the selection process.

### 2.2 Inclusion criteria

In this study, the inclusion and exclusion criteria were developed based on the Population, Intervention, Comparison, Outcomes, Study Design principle. Studies that met the following criteria were included in this meta-analysis: (1) Studies involving HFRS patients diagnosed using one or more of the following criteria: positive serum-specific IgM or a fourfold increase in serum-specific IgG between the acute and recovery phases, detection of hantavirus RNA in the patient’s specimen, or isolation of hantavirus from the patient’s specimen. The diagnostic criteria for AP included serum lipase or amylase levels elevated, three or more times the upper limit of normal, and imaging features of AP on enhanced computed tomography, magnetic resonance imaging, or transabdominal ultrasound. (2) Studies providing data on HFRS patients complicated by AP. (3) Studies with prospective or retrospective, designs, including cohort studies, case–control studies, and cross-sectional studies. (4) Studies reporting author name, publication date, country of origin, the number of HFRS cases, and the incidence of the number of HFRS cases complicated by AP. Studies were excluded if they met the following criteria: (1) Meetings abstracts, case reports, reviews, letters and duplicate publications. (2) Studies that do not provide information on the incidence of HFRS with AP or where this incidence could not be calculated. Two reviewers independently screened the studies based on the inclusion and exclusion criteria. Any discrepancies in study selection are resolved by consulting a third reviewer.

### 2.3 Data extraction

In this study, data extraction was performed by one reviewer who was not involved in the initial literature screening. Another reviewer then checked the data and contacted the authors of the included studies to resolve any missing or incomplete information. The data extracted included the first author details, year of publication, study region, study design, patient demographics (sex and age), total number of HFRS patients and number of patients with HFRS complicated by AP or incidence of HFRS complicated by AP.

### 2.4 Quality assessment of the studies

The methodological quality of the included studies was independently assessed by two reviewers. For cross-sectional studies, the Agency for Healthcare Research and Quality (AHRQ) scale was used (23), which consists of 11 items. Each “yes” response was awarded 1 star (*), with a maximum score of 11 stars. The studies were categorized as follows: 0–3 stars were rated as low quality, 4–7 stars as moderate quality, and 8–11 stars as high quality. For cohort and case–control studies, the Newcastle-Ottawa Evaluation Scale (NOS) (24) was employed, which evaluates studies based on comprising eight items grouped into three categories. The NOS uses a star system, with a maximum of nine stars. The studies were rated as follows: 0–3 stars were rated as low quality, 4–6 stars as moderate quality, and 6–9 stars as high quality.

### 2.5 Statistical analyses

Meta-analysis was conducted using Stata 13.0 software. The pooled incidence rate and 95% confidence interval (CI) were used to evaluate the effect. Heterogeneity among different studies was assessed using Cochran’s *Q* test and the *I^2^* statistic. Heterogeneity was considered present when *p* < 0.05 or *I*^2^ > 50%, in which case a random-effect model was employed to combine the effect estimates. Otherwise, a fixed-effect model was used. Subgroup analysis was performed based on study region, study design, gender, and methodological quality to explore potential sources of heterogeneity. Sensitivity analysis was conducted to evaluate the robustness of the results. Publication bias was assessed using a Funnel plot, Begg’s test, and Egger’s test. A two-tailed *p* value <0.05 was considered statistically significant.

## 3 Results

### 3.1 Study selection and characteristics

**Figure 1:**
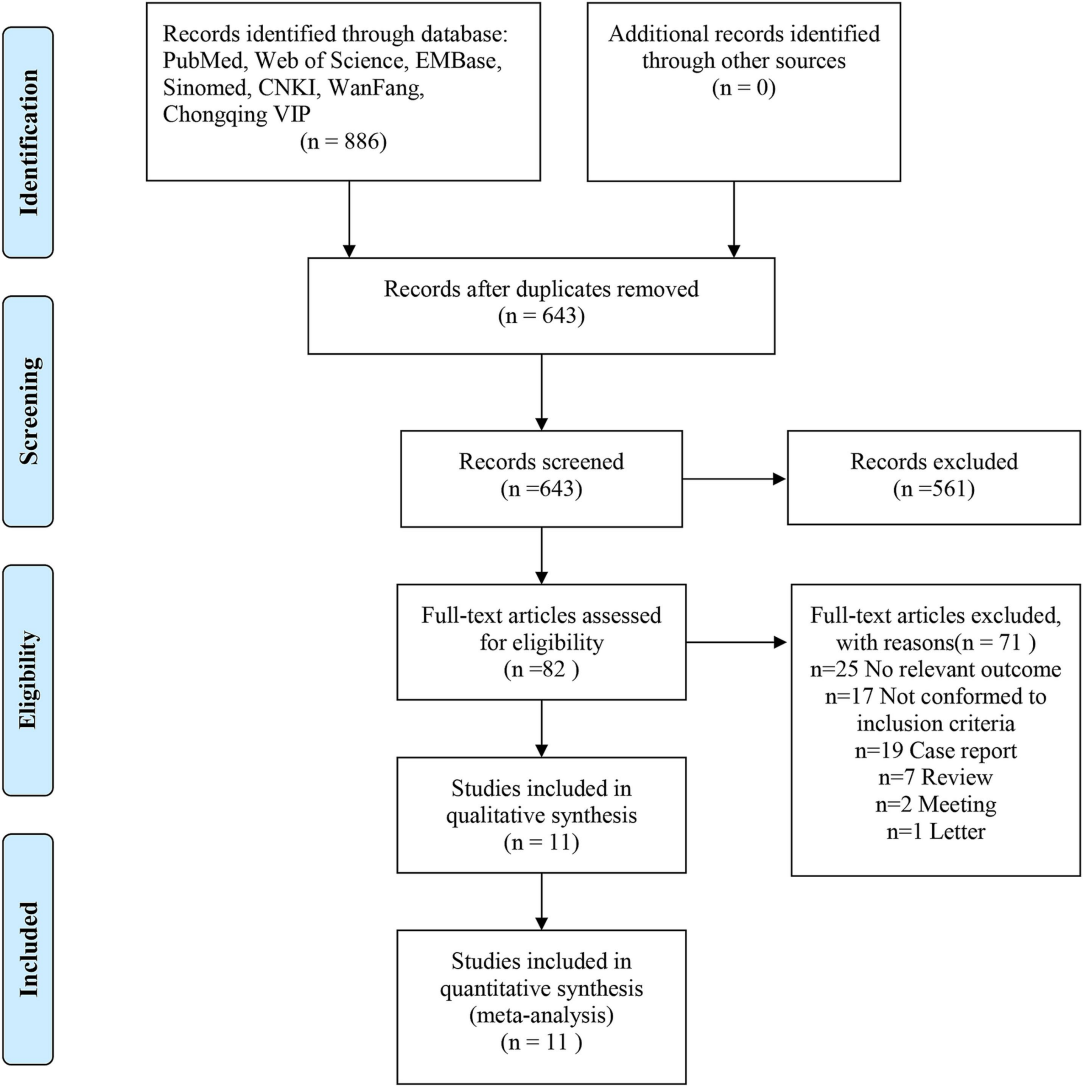
Flow diagram of literature retrieval.

Using the search strategy, a total of 886 relevant studies were identified. After removing duplicates, 643 studies remained. Screening of titles and abstracts led to the exclusion of 561 irrelevant studies, leaving 82 studies for full-text review. Based on the eligibility criteria, 71 studies were excluded, and 11 studies were included in the final meta-analysis (Figure 1).

Among the 11 included studies (20–22, 25–32), seven were published in English and four in Chinese. Geographically, nine studies were from China, 1 from Albania, and one from Slovenia. The analysis included three case–control studies and 8 cross-sectional studies. Ten studies provided the age of the participants, whereas one study did not. Six studies reported mortality data comparing HFRS patients with and without AP, whereas the remaining studies did not provide or were unable to calculate death-related data. After assessing the quality using AHRQ and NOS, nine studies were rated as high quality and two as moderate quality. Overall, the studies included in this meta-analysis were of high methodological quality. The basic characteristics of the included studies are presented in Table 1.

**Table 1:** Characteristics of 11 studies included in this meta-analysis.

ID	First author	Publication year	Country	Research design	Data source	Age	HFRS	HFRS with AP (Death/Alive)	HFRS without AP (Death/Alive)	Quality of literature
1	Bren AF (25)	1996	Slovenia	Cross-sectional study	Data of HFRS from Department of Nephrology University Medical Center during 1983 to 1995.	16–71	33	4 (NA/NA)	29 (NA/NA)	Moderate
2	Zhu Y (26)	2013	China	Cross-sectional study	Data of HFRS from The First Affiliated Hospital of Nanchang University during May 2006 to May 2012.	11–75	218	10 (0/10)	208 (14/194)	High
3	Fan H (21)	2013	China	Cross-sectional study	Data of HFRS complicated with acute pancreatitis from Ningbo first hospital during September 2001to December 2012.	NA	156	12 (1/11)	144 (NA/NA)	Moderate
4	Ren Y (27)	2015	China	Cross-sectional study	The confirmed cases of HFRS in the Third People’s Hospital of Nantong City during October 2012 to December 2014.	20–67	18	2 (0/2)	16 (3/13)	High
5	Puca E (28)	2017	Albania	Cross-sectional study	Data of HFRS from Tirana University Hospital Center, during January 2011 and December 2016	15–59	36	4 (1/3)	32 (3/29)	High
6	Dong X (29)	2018	China	Cross-sectional study	Cases of HFRS diagnosed in the First Affiliated Hospital of Zhengzhou University from January 2010 to December 2016.	Median: 44.70 ± 12.60	55	2 (0/2)	53 (2/51)	High
7	Chen ZM (30)	2018	China	Cross-sectional study	Cases of HFRS diagnosed in the Affiliated Hospital of Shanxi University of Traditional Chinese Medicine from October 2016 to February 2018.	13–76	50	2 (NA/NA)	48 (NA/NA)	High
8	Wang WJ (31)	2020	China	Case–control study	Data of HFRS and ABP cases diagnosed in the First Affiliated Hospital of Wannan Medical College from 2012 to 2018 were collected. Case: HFRS with AP; Control: ABP.	18–85	83	7 (0/7)	76 (NA/NA)	High
9	Guo Q (20)	2021	China	Case–control study	Data of HFRS from the First Affiliated Hospital of Xi’an Jiaotong University during January 2013 to July 2020. Case: HFRS with AP; Control: HFRS without AP.	Median: 46.1 ± 11.5	346	29 (7/22)	317 (7/310)	High
10	Xia Y (22)	2023	China	Case–control study	Cases of HFRS diagnosed in the First Affiliated Hospital of Nanjing Medical University from January 2017 to September 2021 were collected. Case: HFRS with AP; Control: HFRS without AP	Median: 49.1 ± 13.6	109	20 (1/19)	89 (2/87)	High
11	Wang W (32)	2023	China	Cross-sectional study	cases of HFRS diagnosed with HFRS in the First Affiliated Hospital of Wannan Medical College from July 2012 to September 2021.	Median: 49.3 ± 14.8	114	18 (NA/NA)	96 (NA/NA)	High

NA, not applicable.

### 3.2 Overall incidence of HFRS complicated with AP

**Figure 2:**
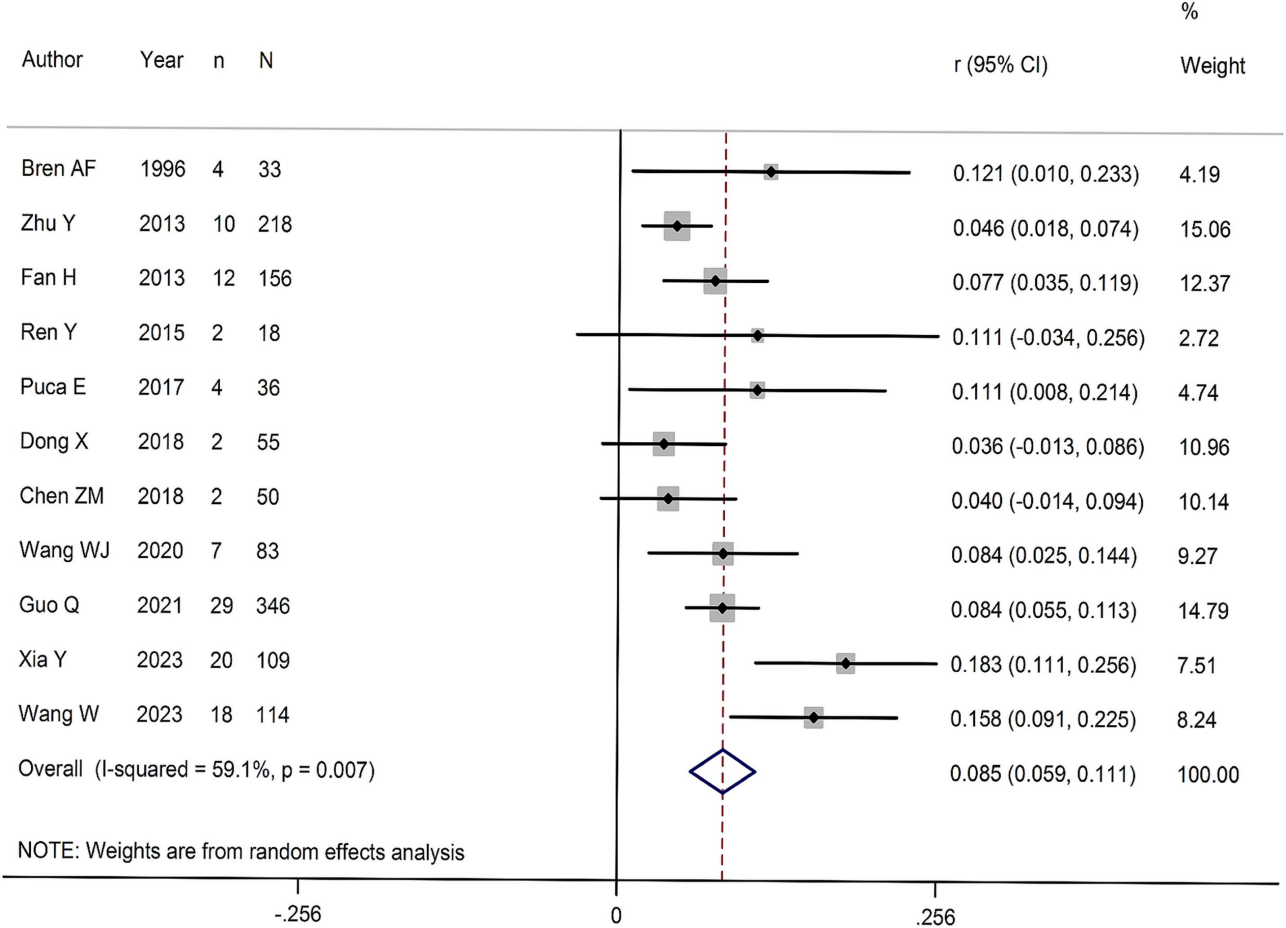
Forest map of the incidence in HFRS patients combined with AP.

A total of 1,218 HFRS patients were reported across the 11 studies, of which 110 patients had AP. The incidence of HFRS combined with AP ranged from 3.6 to 18.3%. Five of the 11 studies report AP incidence of >10%, and 6 studies reporting an AP incidence of ≤10%. Significant heterogeneity was observed among the studies (*Q* = 24.44, *p* < 0.001; *I^2^* = 59.1%). Therefore, a random effects model was used to calculate the pooled effect size (Figure 2). The overall incidence of HFRS complicated with AP was 8.5% (95% *CI* for *r* 5.9–11.1%; *Z* = 6.40, *p* = 0.001).

### 3.3 Subgroup analyses

Subgroup analysis was conducted based on gender, sample size, region, study design, and methodological quality (Table 2). The incidence of HFRS with AP was 9.4% (95% *CI* for *r* 5.7–13.1%) in males and 10.8% (95% *CI* for *r* 5.6–16.0) in females. The incidence of HFRS with AP was 6.2% (95% *CI* for *r* 3.4–9.0%) in studies with a sample sizes of <100, and 9.9% (95% *CI* for *r* 5.9–14.0%) in studies with a sample size of ≥100. Regarding regional distribution, the incidence of HFRS with AP was 8.2% (95% *CI* for *r* 5.4–11.0%) in Asia and 11.6% (95% *CI* for *r* 4.0–19.1%) in Europe. The incidence of HFRS with AP was 11.0% (95% *CI* for *r* 5.6–16.4%) in case–control studies and 7.3% (95% *CI* for *r* 4.4–10.3%) in cross-sectional studies. Regarding different methodological quality grades, the incidence of HFRS with AP was 8.6% (95% *CI* for *r* 5.5–11.7%) in high quality studies and 8.2% (95% CI for *r* 4.3–12.2%) in moderate quality studies.

**Table 2:** The incidence of HFRS complicated with AP in different subgroups.

Subgroup	No. of studies	*r*(%)	95%*CI* for *r*	Heterogeneity		Begg’s test		Egger’s test		Meta-regression	
				*I*^2^(%)	*p*	*Z*	*p*	*t*	*p*	*t*	*p*
Gender											
Male	6	9.4	5.7–13.1	61.1	0.017	1.05	0.293	2.25	0.060	0.460	0.656
Female	6	10.8	5.6–16.0	29.5	0.225	0.49	0.624	1.89	0.115		
Sample size											
<100	6	6.2	3.4–9.0	0.0	0.466	1.69	0.091	2.05	0.400	0.910	0.386
≥100	5	9.9	5.9–14.0	78.8	0.001	1.96	0.50	4.92	0.039		
Location											
Asia	9	8.2	5.4–11.0	65.5	0.003	1.88	0.061	1.97	0.180	0.670	0.520
Europe	2	11.6	4.0–19.1	0.0	0.896	1.00	0.317	2.28	NA		
Research design											
Case–control study	3	11.0	5.6–16.4	68.7	0.041	1.57	0.117	2.76	0.478	−1.090	0.305
Cross-sectional study	8	7.3	4.4–10.3	49.0	0.056	1.48	0.138	1.75	0.106		
Quality of study											
Medium	2	8.2	4.3–12.2	0.0	0.466	1.00	0.317	1.82	NA	−0.080	0.935
High	9	8.6	5.5–11.7	66.3	0.003	1.67	0.095	1.25	0.169		

NA, not applicable. In the meta-regression analysis, the reference groups were Male, <100, Asia, Case–control study, Medium, respectively.

To investigate the heterogeneity among the included studies, statistical comparisons were made between the subgroups. Meta-regression analysis results were present in Table 2, and no statistically significant differences were observed among the various subgroups. The results of the subgroup analysis and meta-regression suggesting a high degree of homogeneity across all included studies. Nevertheless, unrecognized clinical and methodological heterogeneity, or other unknown factors, may still exist. Given the relatively limited number of studies included in this analysis, the findings should be interpreted with caution.

### 3.4 Publication bias

**Figure 3:**
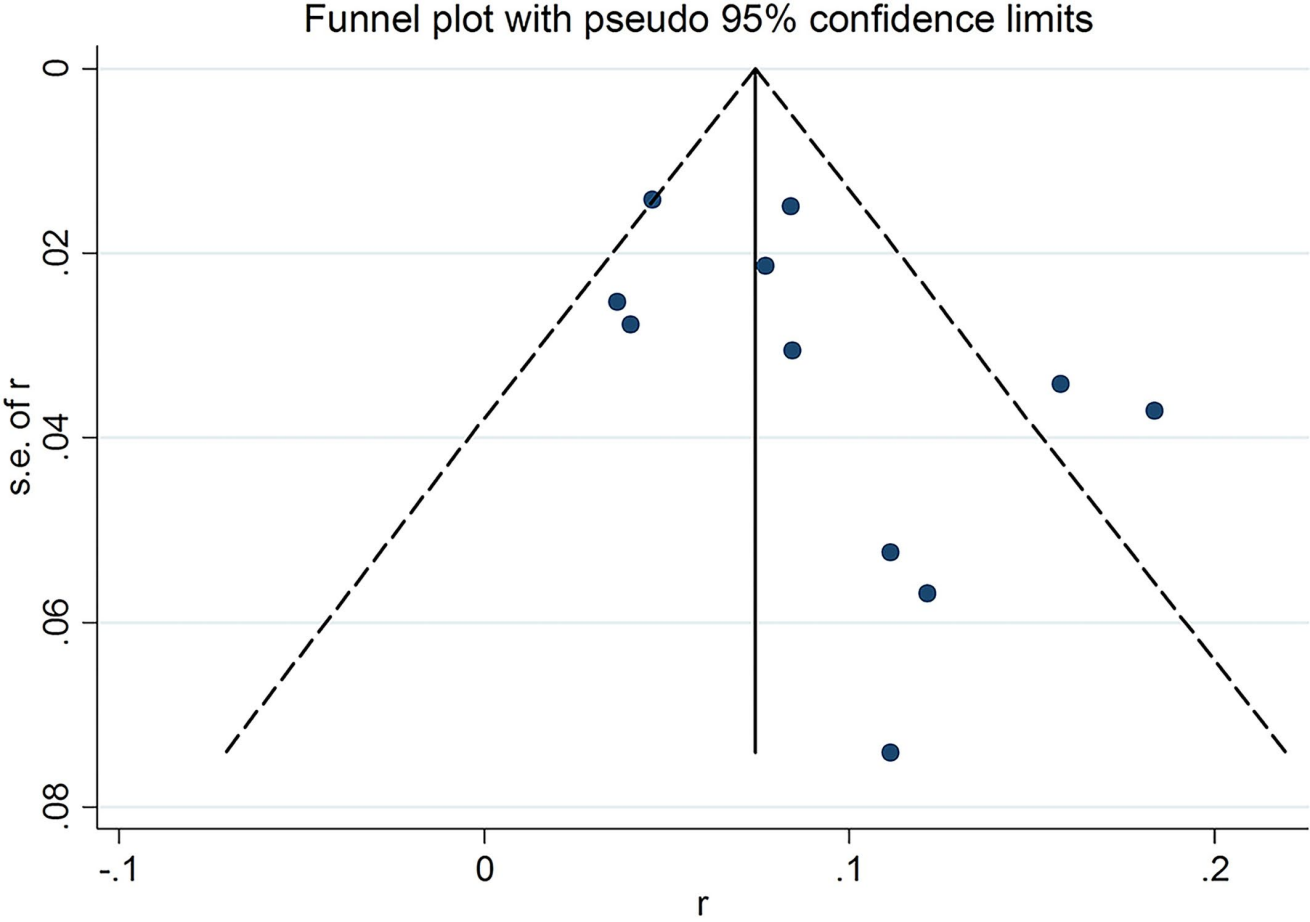
Funnel plot of the incidence in HFRS patients combined with AP.

To assess potential publication bias, a funnel plot was generated, which visually showed an asymmetrical distribution of the included studies (Figure 3). Despite this, statistical tests, including Begg’s test (*Z* = 1.71, *p* = 0.087) and Egger’s test (*t* = 1.82, *p* = 0.102), revealed no significant evidence of publication bias. Altogether, both subjective and objective evaluations suggested that publication bias was not present in this study.

### 3.5 Sensitivity analysis

**Figure 4:**
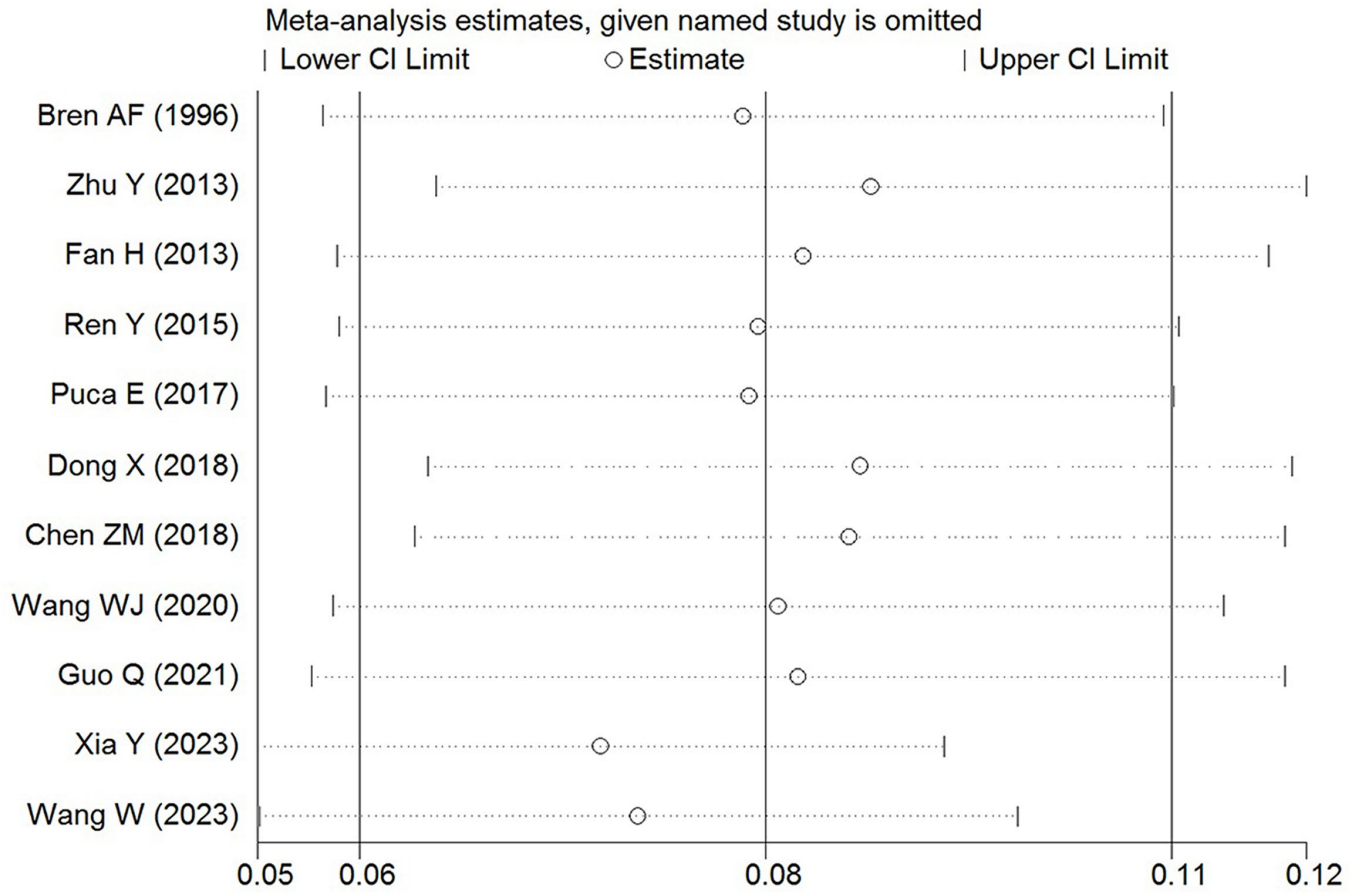
Sensitivity analysis of incidence in HFRS patients combined with AP.

Sensitivity analysis was conducted to evaluate robustness and stability of the results. By systematically excluding each study and recalculating the pooled effect size, we determined that the overall incidence of HFRS complicated with AP remained stable after the exclusion of any individual study. This confirms the reliability of the findings (Figure 4).

### 3.6 Risk of death in HFRS patients with AP

**Figure 5:**
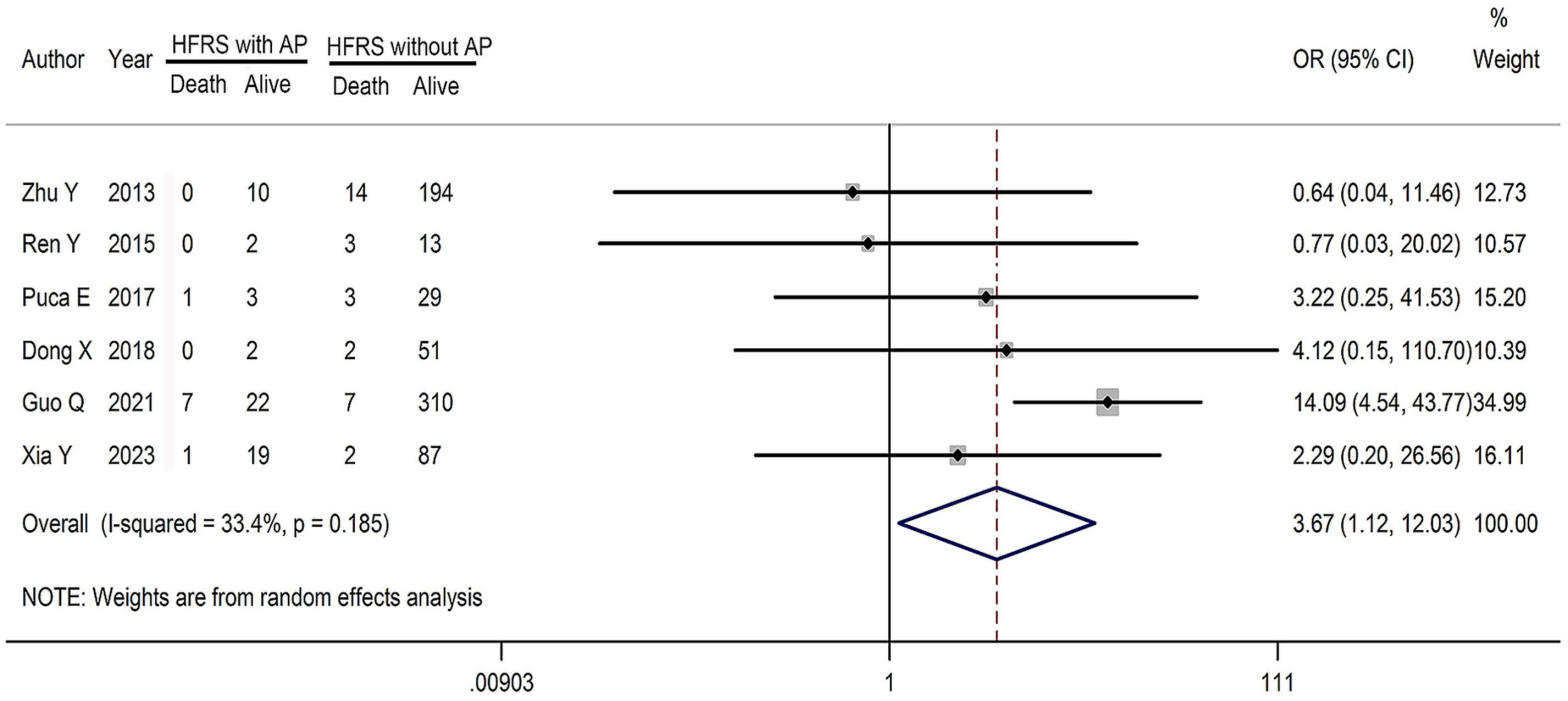
Forest plots of mortality risk in HFRS patient combined with AP.

This study also assessed the mortality risk in HFRS patients with AP compared with those without AP, using mortality as the outcome indicator. Six studies reported mortality data, with the mortality rate for HFRS combined with AP ranging from 0.0 to 25.0%, while that for HFRS without AP ranging from 2.2 to 18.8%. The effect sizes (OR) among the various studies ranged from 0.639 to 14.091. No significant heterogeneity was found (*Q* = 7.51, *p =* 0.185 > 0.05; *I^2^* = 33.4%; Figure 5); therefore, a fixed-effect model was used. The pooled effect size (OR) was 3.668 (95%*CI* for *OR* 1.112–12.031, *Z* = 2.14, *p* = 0.032 < 0.05). This indicates that the mortality risk in HFRS patients with AP was significantly higher than that in patients without AP.

## 4 Discussion

Based on the current literature search, we believe that this is the first systematic meta-analysis to comprehensively analyze the occurrence of HFRS combined with AP. In this study, the incidence of HFRS combined with AP was 8.5% (95% *CI* for *r* 5.9–11.1%), which, while not exceedingly high, underscores the significance of clinical vigilance in these cases. The pathogenesis of AP in HFRS patients may involve multiple factors. Hantavirus may directly damage pancreatic tissues, while the release of inflammatory mediators and immune system dysregulation may exacerbate pancreatic injury. Clinicians should be particularly alert to the potential for AP in HFRS patients who present with abdominal pain, a history of alcohol consumption, and signs such as abnormal coagulation hypoalbuminemia, thrombocytopenia, elevated lymphocyte ratios, or high levels of proteinuria (33, 34).

The incidence of AP in HFRS patients was 9.4% in males and 10.8% in females, aligning with prior research that also found no marked gender disparity in AP incidence (35, 36). However, the incidence of AP varied depending on the sample size. The studies with sample size of ≥100 reported a higher incidences of AP than studies with a sample size of <100. Regarding regional distribution, the incidence of AP in HFRS patients was higher in Europe than in Asia. However, as the present study only included two European datasets, the sample size may have been too small to draw definitive conclusions. Generally, the larger the sample size, the more stable the results of the relative number calculation (37, 38). Therefore, larger studies across various regions are needed to refine the understanding of geographic variability in AP incidence among HFRS patients. The incidence of AP in case–control studies was higher than cross-sectional studies. This may be due to the admission bias and detected syndrome bias in the selection of subjects in case–control studies, resulting in more reported cases of HFRS with AP (39).

In addition to the primary findings, an analysis of six studies reporting on mortality revealed that HFRS patients with AP had a higher significantly risk of mortality than those without AP (*OR* = 3.668, 95% *CI* for *OR* 1.112–12.031). Two potential reasons for this increased risk have been proposed. First, HFRS patients may experience more severe inflammation, leading to more frequent liver dysfunction and increasing the likelihood of developing into critical or severe cases (40). Second, HFRS complicated with AP is often misdiagnosed due to its nonspecific clinical manifestations and failure to timely treatment increases the mortality risk (41). Because most patients with HFRS exhibit nonspecific clinical manifestations, their typical symptoms are primarily characterized by gastrointestinal system symptoms, such as fever, nausea, vomiting, abdominal pain, and abdominal distension, that overlap with gastrointestinal disorders such as AP. This diagnostic overlap can delay appropriate treatment, further elevating the mortality risk. Consequently, clinicians should exercise caution in ruling out the possibility of AP in HFRS patients presenting with fever and abdominal pain.

## 5 Limitations

The study has several limitations in this study. First, only published studies were included in the meta-analysis, which could result in selection bias, as unpublished or unreported studies were not considered. Second, the studies included in this meta-analysis were mostly from China. This geographic concentration may not fully represent the geographic sample. Third, other potential factors affecting the incidence AP in patients, such as age, lifestyle, or underlying health conditions, could not be fully investigated due to the limited number of studies and the variable quality of available data.

## 6 Conclusion

The overall incidence of HFRS complicated with AP was 8.5%, while not high, which is concerning because of the significantly increased risk of mortality for HFRS patients when AP is present. In the future, additional large-scale prospective studies are warranted to determine the incidence of HFRS with AP and the associated mortality risk.
